# Adherence of private health system hospitals to dissemination of outcomes according to the Global Reporting Initiative (GRI) model

**DOI:** 10.1590/S1679-45082017GS3989

**Published:** 2017

**Authors:** Celso Machado, Robson Danúbio da Silva César, Maria Tereza Saraiva de Souza

**Affiliations:** 1 Centro Universitário das Faculdades Metropolitanas Unidas Centro Universitário das Faculdades Metropolitanas Unidas São PauloSP Brazil Centro Universitário das Faculdades Metropolitanas Unidas, São Paulo, SP, Brazil.; 2 Centro Universitário da Fundação Educacional Inaciana “Padre Sabóia de Medeiros” São PauloSP Brazil Centro Universitário da Fundação Educacional Inaciana “Padre Sabóia de Medeiros”, São Paulo, SP, Brazil.

**Keywords:** Sustainable development, Health facilities, Global Reporting Initiative, Governance/organization & administration, Indicators

## Abstract

**Objective:**

To verify if there is an analogy between the indicators of the Global Reporting Initiative adopted by hospitals in the private healthcare system.

**Methods:**

Documentary research supported by reports that are electronically available on the website of the companies surveyed.

**Results:**

The organizations surveyed had a significant adherence of their economic, social and environmental indicators of the model proposed by the Global Reporting Initiative, showing an analogous field of common indicators between them.

**Conclusion:**

There is similarity between the indicators adopted by companies, but one of the hospitals analyzed had a greater number of converging indicators to Global Reporting Initiative.

## INTRODUCTION

Access to health by Brazilians can occur through the National Unified Healthcare System (SUS – *Sistema Único de Saúde*) and is a responsibility of the government, or in a supplementary manner by private care. Among the players that act in the private healthcare system, hospitals that provide a broad spectrum of care to the citizen stand out.^(^^1^^)^

Besides developing the important function of providing health care to citizens, the hospitals are subject to market competitiveness laws. To meet the relation demands with society, employees and shareholders in corporative governance, have a set of good practices that align interests with the purpose of preserving and optimizing the value of the organization, facilitating access to resources.^(^^2^^)^ Among these good practices is disclosure, in which the organizations should report their social, environmental, and economic performance, in a transparent fashion.

According to the Brazilian Institute of Corporate Governance, disclosure - which comes from the need to enable transparency of results of the activities executed by the company, consists in one of the items pointed out as good practices*.*^(^^2^^)^ Despite the different grounds that involve disclosure, the most recent approach positions it as an act of dissemination of quantitative and qualitative information, whether formally or informally, to help users as to the opportunities and risks related to the organization.^(^^3^^)^ The company, in increasing its level of voluntary reporting, establishes a scenario in which it is distinguished from the others, since it reports more than is required by law or by market rules.^(^^4^^)^

The importance of the companies divulging their indicators is pointed out due to the fact that the companies showimg a greater and better degree of reputation, also show a greater measure of voluntary disclosure.^(^^5^^)^

In Brazil, the preparation of sustainability or social-environmental reports is not mandatory by law. The companies that expose their information can be characterized as practicing voluntary disclosure. Additional dissemination of information may benefit the company, leading to enhanced corporate reputation, improved performance and strive for financial returns resulting from this practice.^(^^6^^)^

Due to the benefits of information disclosure, it is possible to infer that all the companies would be interested in expanding the availability of their data to society. However, many companies still position themselves timidly or are neglectful in dissemination of their information. The following are reasons for non-disclosure: lack of incentive for the manager, lack of knowledge about information, and nonexistence of the information on the part of the company.^(^^7^^)^

More recent studies indicate that there is a greater demand of the stakeholders as to disclosure of financial, environmental, and social information,^(^^8^^)^ a condition that reinforces the understanding of the importance of transparency of information as a component for sustainability and for corporative governance.^(^^4^^)^

In the current competitive environment, the search for the best practices or differentiation in the market may lead to the company being chosen among the rest by investors. When a company stands out, its competitors seek to be equal or superior to it.^(^^9^^)^ Based on its disclosure, in which greater transparency implies a better reputation, the market chooses the companies.^(^^10^^)^

Although the reports incorporate the social, environmental, and economic dimensions, these do not appear homogeneously. There are different intensities of adherence in the disclosure of information available in the sustainability reports of the companies, according to the classification of the Global Reporting Initiative (GRI)^(^^11^^)^ indicators. Adherence to social disclosure reaches 71%, while environmental disclosure is 57%.^(^^12^^)^ Companies have reported their social practices more intensely, geared towards the local community and human resource management, comparatively to the environmental practices related to protection and preservation of the environment. The sustainability report is therefore established as an important communication tool of social, environmental, and economic performance of the companies.^(^^12^^)^

## OBJECTIVE

To identify if there is an analogy between the indicators adopted by hospitals of the private healthcare system. To reach this objective, besides this introductory chapter that contains the theoretical reference, the study presents other methods used, the results obtained, discussion, and conclusion.

## METHODS

This is a qualitative investigation, based on a documental study. Qualitative research adapts to the studies geared towards showing the complexity of a problem, as well as classifying and analyzing dynamic processes.^(^^13^^)^

We analyzed reports available electronically on the websites of the companies investigated. The search for performance reports showed a low number of healthcare organizations that use this practice. The study covered hospitals listed as the best in 2015 (http://exame.abril.com.br/negocios/as-18-campeas-por-setor-em-melhores-e-maiores-2015/). This methodological option was due to the fact of identifying the best hospitals that potentially have greater interest in and/or resources for disclosing their performance. Among the reports identified, we prioritized the data that could be compared. Thus, the following choice criteria were established for the companies compared: companies of the same sector and with similar economic size, and that published the performance report as per GRI criteria.

Three hospitals were selected, identified by fictitious names: Hospital-AE, founded in São Paulo, on June 4, 1955; Hospital-AC founded in São Paulo, on April 23, 1953; and Hospital-SL, officially opened on August 15, 1965, in the city of São Paulo.

The understanding and coordination of the relevant information are based on the Content Analysis*,* which is a technique based on systematic procedures and which verifies the content of texts and indicators in order to infer, both in knowledge and in conditions of message production and reception.^(^^14^^)^

## RESULTS

As a documental base, we used the reports published by the healthcare organizations in 2014. Table 1 presents the adherence of the healthcare organizations to the GRI indicators in the economic, social, and environmental aspects.

**Table 1:** Adherence of the healthcare organizations to the Global Reporting Initiative indicators

Companies	Indicators								

	Economic			Social			Environmental		
		
	Adheres (%)	Does not adhere (%)	Did not present (%)	Adheres (%)	Does not adhere (%)	Did not present (%)	Adheres (%)	Does not adhere (%)	Did not present (%)
Hospital-AE	73.5	0.0	26.5	55.6	0.0	44.4	62.5	0.0	37.5
Hospital-AC	32.4	17.6	50.0	33.3	33.3	33.3	35.4	16.7	47.9
Hospital-SL	38.2	0.0	61.8	44.4	0.0	55.6	33.3	0.0	66.7
Mean	48.0	5.9	46.1	44.4	11.1	44.4	43.8	5.6	50.7

The percentage expresses the adherence index of the financial institution’s report relative to that proposed by GRI.

The economic dimension was better covered by the GRI indicators, with a mean adherence of 48.0%. Next, the social dimension, with 44.4%, and the environmental, with 43.8%. Hospital-AC was the only one to present indicators that did not adhere to GRI in the three dimensions of sustainability. Hospital-AE presented better adherence in each of the three categories.

Table 2 shows the classification of the social indicators of the healthcare organizations.

**Table 2:** Classification of the social indicators of healthcare organizations

Social indicators	Hospital-AE	Hospital-AC	Hospital-SL
Assessment of suppliers regarding Human Rights	2	2	-
Assessment of suppliers regarding impacts on society	-	1	-
Assessment of suppliers in work practices	-	2	-
Fighting corruption	3	1	-
Local communities	2	-	2
Diversity and equality of opportunities	1	1	-
Employment	3	1	3
Remuneration equality between women and men	1	-	-
Investments	-	-	1
Mechanisms for complaints and appeals relative to impacts on society	1	-	-
Mechanisms for complaints and appeals relative to work practices	1	-	-
Non-discrimination	-	-	1
Privacy of the client	1	-	-
Work relations	1	-	-
Labeling of products and services	3	1	1
Health and safety of the client	2	1	2
Health and safety at work	4	4	3
Forced labor or analogous to slavery	1	-	-
Child labor	1	-	-
Training and education	3	3	3

The volume and the diversity of aspects were greater in the economic and environmental dimensions. Hospital-AE had 30 indicators, while Hospital-AC had 17, and Hospital-SL, 16. Hospital-AE was the organization that most adhered and divulged their social indicators, with almost double the number of indicators relative to its competitors. The classification of the environmental indicators of the healthcare organizations is on table 3.

**Table 3:** Classification of the environmental indicators of healthcare organizations

Environmental indicators	Hospital-AE	Hospital-AC	Hospital-SL
Water	3	3	3
Assessment and environment of suppliers	2	1	-
Effluents and waste	5	3	3
Emissions	7	-	3
Energy	5	3	4
Materials	2	-	-
Mechanisms for complaints and appeals relative to environmental impacts	1	-	-
Products and services	-	1	-

The attention to the environmental dimensions was noted in all healthcare organizations. Hospital-AE showed the greatest quantity of indicators 25, standing out in relation to the other two. Hospital-SL appeared in second place, with 13 indicators, and Hospital-AC had 11. Table 4 presents the classification of the economic indicators of healthcare organizations.

**Table 4:** Classification of the economic indicators of healthcare organizations

Economic indicators	Hospital-AE	Hospital-AC	Hospital-SL
Economic performance	4	1	2
Indirect economic impacts	0	2	2
Market presence	1	0	0

Hospital-AC and Hospital-SL did not display market presence, while Hospital-AE did not show indirect economic impacts. The total number of indicators per healthcare organizations was: Hospital-AE with 117, Hospital-AC with 85, and Hospital-SL with 69. Considering only the indicators belonging to GRI, this was the profile: Hospital-AE with 60, Hospital-AC with 33, and Hospital-SL with 31.

The total number of sustainability indicators in its three dimensions was superior in Hospital-AE, while in the other healthcare organizations they are very close as to the number of indicators reported. This profile shows greater interest of Hospital-AE in showing its performance in the form of indicators, as per the GRI recommendation.

## DISCUSSION

The comparison between the sustainability reports of the three companies allowed the combined analysis of the data and revealed the interest of the healthcare organizations in publishing their performance in the form of GRI-adhering indicators. The dissemination of the reports corroborates the approach of interest of the organizations in disseminating their data,^(^^12^^)^ enabling the stakeholders to understand, even if partially, about sustainability of the companies.^(^^12^^)^

The larger volume of information reported by Hospital-AE may result in corporate benefits, especially those related to the reputation of the organization.^(^^6^^)^ Hospital-AE began publishing their report in 2006, while the other hospitals analyzed began this process five years later. This scenario can be explained by the approach in which the competitors feel pressured to disclose their data to the extent that one of the players stands out for publishing reports that show a good performance of the indicators.^(^^9^^)^ The fact that they have published their indicators for a longer period of time and the larger volume of information available on Hospital-AE, positions it with distinction,^(^^4^^)^ and leads to a better reputation of the institution.^(^^5^^)^ The data obtained does not allow the identification of whether the smaller volume of information made available by the other two organizations is related to limiting factors.^(^^7^^)^

The short longevity of the reports from the hospitals corroborates the understanding that points to the disclosure of performance, on the part of the companies, including sustainable indicators as a recent action.^(^^8^^)^

The three organizations presented identical profiles of proportionality of indicators. The social indicators were the ones that appeared in greatest volume, followed by the environmental and economical, a condition similar to that identified in other investigations.^(^^11^^)^

We observed a set of 20 themes that covered 63 social indicators used by the companies. The categories that stood out were those of health and safety at work, with 11 indicators, and training and education, with 9, considering the three healthcare organizations. These categories tend to have a close relation among them, since the professional activities carried out demand the need for health care and safety at work.

The environmental indicators were distributed into eight categories, but concentrated in four of them, namely: energy with 12 indicators, effluents and waste with 11 indicators, emissions with 10 indicators, and water with 9 indicators, considering the three healthcare organizations. These indicators point to two attention groups: the first geared towards optimization of resources (energy and water), and the second, towards monitoring and mitigation of the impact of their activities (effluents and waste, and emissions). This indicates attention needed for specific environmental themes for the healthcare organizations.

The adherence of Hospital-AE to the disclosure of its environmental indicators proved superior to the other organizations. Hospital-AE reported 117 indicators, while Hospital-AC has 85, and Hospital-SL, 69. Particularly adressing the indicators that adhere to the model proposed by GRI, we observe a predominance of Hospital-AE, with 63 complying indicators, a volume superior to the sum of the other two hospitals analyzed.

## CONCLUSION

The comparison of the healthcare organizations active in the same segment shows that there was an analogy among the indicators adopted by the companies. We identified the analogy of indicators used by the three healthcare organizations, both for environmental and for social and economic sustainability. Additionally, we identified the similarity among the indicators used by the healthcare organizations in the dimensions profile and governance.

The sustainability reports of the hospitals analyzed had indicators in all dimensions of sustainability, but with different adherence intensity. The social dimension indicators appeared in greater volume and in the sequence, the environmental and economic indicators, with smaller quantities.

## References

[1] 1. Azevedo AL, Costa AM. [The narrow entrance door of Brazil’s National Health System (SUS): an evaluation of accessibility in the Family Health Strategy]. Interface (Botucatu). 2010;14(35):797-810. Portuguese.

[2] 2. Instituto Brasileiro de Governança Corporativa (IBGC). Código das melhores práticas de governança corporativa. 4a ed. São Paulo: IBCC; 2009.

[3] 3. Carmona P, Fuentes C, Ruiz C. Risk disclosure analysis in the corporate governance annual report using fuzzy-set qualitative comparative analysis. Rev Adm Companies. 2016;56(3):342-52.

[4] 4. Jermias J, Lindawati G. The impact of board capital and board characteristics on firm performance. Br Account Rev. 2014;46(2):135-53.

[5] 5. Cruz CV, Lima GA. [Corporate reputation and disclosure level in open capital Brazilian companies]. Rev Universo Contábil. 2010;6(1):85-101. Portuguese.

[6] 6. Machado Filho CP. Responsabilidade social e governança: o debate e as implicações. São Paulo: Pioneira; 2006.

[7] 7. Dumay J. A critical reflection on the future of intellectual capital: from reporting to disclosure. J Intellectual Capital. 2016;17(1):168-84.

[8] 8. Aburaya RK. The Relationship between Corporate Governance and Environmental Disclosure: UK Evidence [Internet]. Durham: Durham University; 2012 [cited 2017 June 2]. Available from: http://etheses.dur.ac.uk/3456/1/PhD_Thesis.pdf?DDD2+

[9] 9. Calve A, Nossa V, Pagliarussi MS, Teixeira AJ. [A study of corporate governance in hospitals charitable in Espírito Santo]. Rev Univ Contábil. 2013; 9(4):128-43. Portuguese.

[10] 10. Gomes PH, De Luca MM, Vasconcelos AC, Ponte VM. Fatores determinantes do disclosure voluntário sob o enfoque da sustentabilidade: uma análise das companies dos países do BRIC. Rev Gestão Social Ambient. 2015;9(2):70-87.

[11] 11. Cardoso VI, De Luca MM, Gallon AV. [Corporate Reputation and the Socio-environmental Disclosure of Brazilian Firms]. Rev Contabilidade Gestão Governança. 2014;17(2):26-35. Portuguese.

[12] 12. Benites LL, Polo EF. [Sustainability as business strategic: the corporate governance and application of the triple bottom line in masisa]. Rev Adm UFSM. 2013;6(Ed Espec):195-210. Portuguese.

[13] 13. Richardson RJ. Pesquisa social: métodos e técnicas. 3a ed. rev. e ampl. São Paulo: Atlas; 2007.

[14] 14. Bardin L. Análise de conteúdo. Lisboa: Edições 70; 2009.

