# Supplementary material for: Adherence of private health system hospitals to dissemination of outcomes according to the Global Reporting Initiative (GRI) model
Source: Einstein (Sao Paulo). 2017 Jul-Sep;15(3):344–8. doi: 10.1590/S1679-45082017GS3989 (PMC5823050; doi:10.1590/S1679-45082017GS3989)
Supplement: Translation

# Translation: Adesão hospitalar do sistema suplementar de saúde à divulgação de resultados segundo modelo *Global Reporting Initiative* (GRI)

**Authors:** Celso Machado, Robson Danúbio da Silva César, Maria Tereza Saraiva de Souza

**Affiliations:** 1 São PauloSP Brasil Centro Universitário das Faculdades Metropolitanas Unidas, São Paulo, SP, Brasil.; 2 São PauloSP Brasil Centro Universitário da Fundação Educacional Inaciana “Padre Sabóia de Medeiros”, São Paulo, SP, Brasil.

**Keywords:** Desenvolvimento sustentável, Instituições de saúde, Global Reporting Initiative, Governança/organização & administração, Indicadores

## Abstract

**Objetivo:**

Identificar se há analogia entre indicadores do *Global Reporting Initiative* adotados por hospitais do sistema suplementar de saúde.

**Métodos:**

Investigação documental apoiada em relatórios disponibilizados por via eletrônica no sítio das empresas pesquisadas.

**Resultados:**

As instituições pesquisadas possuíam significativa aderência de seus indicadores econômicos, sociais e ambientais ao modelo proposto pelo *Global Reporting Initiative*, estabelecendo um campo análogo de indicadores comuns entre elas.

**Conclusão:**

Há analogia entre os indicadores adotados pelas empresas, porém um dos hospitais possuía um número maior de indicadores convergentes ao *Global Reporting Initiative*.

## INTRODUÇÃO

O acesso à saúde pelos brasileiros pode ocorrer por meio do Sistema Único de Saúde (SUS), de responsabilidade governamental, ou de forma suplementar, pela iniciativa privada. Dentre os atores que atuam na saúde suplementar, destacam-se os hospitais, que proporcionam um amplo espectro de atendimentos ao cidadão.^(^^1^^)^

Além de desenvolverem a importante função social de proporcionar saúde ao cidadão, os hospitais estão sujeitos às leis da concorrência do mercado. Para o atendimento adequado das demandas de relacionamento para com a sociedade, os colaboradores e, ainda, os acionistas, identifica-se, na governança corporativa, um conjunto de boas práticas, que alinham interesses com a finalidade de preservar e otimizar o valor da organização, facilitando seu acesso aos recursos.^(^^2^^)^ Dentre estas boas práticas, identifica-se o *disclosure*, no qual as instituições devem divulgar, de forma transparente, seu desempenho social, ambiental e econômico.

Segundo o Instituto Brasileiro de Governança Corporativa, *disclosure*, que advém da necessidade de possibilitar a transparência dos resultados decorrentes das atividades executadas pela empresa, consiste em um dos itens apontados como boas práticas*.*^(^^2^^)^ Apesar das diferentes bases que envolvem o *disclosure*, a abordagem mais recorrente o posiciona como ato de divulgação de informações quantitativas e qualitativas, de maneira formal ou informal, para auxiliar os usuários quanto às oportunidades e aos riscos referente à empresa.^(^^3^^)^ A empresa, ao aumentar seu nível de divulgação voluntariamente, estabelece um cenário em que se distingue das demais, por relatar além do exigido por lei ou pelas regras do mercado.^(^^4^^)^

A importância da divulgação dos indicadores por parte das empresas é destacada diante do fato de se ter identificado que as empresas que apresentam um maior e melhor grau de reputação apresentam também uma quantidade maior de *disclosure* voluntário.^(^^5^^)^

No Brasil, a elaboração de relatórios de sustentabilidade ou socioambientais não é obrigatória por lei. As empresas que divulgam suas informações podem ser caracterizadas como praticantes do *disclosure* voluntário. A divulgação adicional de informações pode beneficiar a empresa, resultando no aumento de sua reputação corporativa, que a leva a aprimorar seu desempenho e almejar retornos financeiros decorrentes da prática.^(^^6^^)^

Por conta dos benefícios de divulgação da informação, seria possível inferir que todas as empresas estariam interessadas em ampliar a disponibilização dos dados para a sociedade. No entanto, muitas empresas ainda se posicionam de forma tímida ou são omissas na divulgação de suas informações. Os motivos para a não divulgação são os seguintes: falta de incentivo para o gestor, falta de conhecimento da informação, e não existência da informação por parte da empresa.^(^^7^^)^

Estudos mais recentes indicam que há uma maior exigência dos *stakeholders* em relação à divulgação de informações financeiras, ambientais e sociais,^(^^8^^)^ condição que reforça o entendimento da importância da transparência de informações como componente para a sustentabilidade e para a governança corporativa.^(^^4^^)^

No atual ambiente competitivo, a busca por melhores práticas ou diferenciação no mercado pode fazer com que a empresa seja escolhida dentre as demais pelos investidores. Quando uma empresa se destaca, seus concorrentes buscam se igualar a ela ou superá-la.^(^^9^^)^ Com base em seu *disclosure*, no qual maior transparência implica em melhor reputação, o mercado escolhe as empresas.^(^^10^^)^

Apesar dos relatórios incorporarem as dimensões social, ambiental e econômica, estes não aparecem de forma homogênea. Há diferentes intensidades de aderência no *disclosure* de informações disponíveis nos relatórios de sustentabilidade das empresas, de acordo com a classificação dos indicadores da *Global Reporting Initiative* (GRI).^(^^11^^)^ A aderência ao *disclosure* social chega a 71%, enquanto o *disclosure* ambiental a 57%.^(^^12^^)^ As empresas têm divulgado com mais intensidade suas práticas sociais, voltadas para a comunidade local e gestão de recursos humanos, comparativamente às práticas ambientais, relacionadas à proteção e à preservação do meio ambiente. O relatório de sustentabilidade estabelece-se, então, como importante ferramenta de comunicação do desempenho social, ambiental e econômico das empresas.^(^^12^^)^

## OBJETIVO

Identificar se há uma analogia entre os indicadores adotados por hospitais do sistema suplementar de saúde. Para o atendimento deste objetivo além deste capítulo introdutório que contém o referencial teórico, o estudo apresenta os métodos empregados, os resultados obtidos, a discussão e a conclusão.

## MÉTODOS

Pesquisa qualitativa, apoiada em um estudo documental. A pesquisa qualitativa se adequa aos estudos voltados a mostrar a complexidade de um problema, bem como classificar e analisar processos dinâmicos.^(^^13^^)^

Foram consultados relatórios disponibilizados por via eletrônica no sítio das empresas pesquisadas. A busca de relatórios de desempenho dos hospitais apontou um baixo número de instituições de saúde que exercem esta prática. A investigação abrangeu hospitais listados como melhores de 2015 (http://exame.abril.com.br/negocios/as-18-campeas-por-setor-em-melhores-e-maiores-2015/). Esta opção metodológica deveu-se ao fato de identificar que os maiores hospitais são os que potencialmente têm maior interesse e/ou recursos para divulgar seu desempenho. Dentre os relatórios identificados, priorizaram-se os dados passíveis de comparação. Assim, foram estabelecidos os seguintes critérios de escolha das empresas de comparação: empresas do mesmo setor e de porte econômico semelhante, e que publicassem o relatório de desempenho segundo os critérios da GRI.

Foram selecionados três hospitais, identificados por nomes fictícios: Hospital-AE, fundado em São Paulo em 4 de junho de 1955; Hospital-AC fundado em São Paulo em 23 de abril de 1953; e Hospital-SL inaugurado oficialmente em 15 de agosto de 1965 na cidade de São Paulo.

O entendimento e a coordenação das informações relevantes se fundamentaram na Análise de Conteúdo, que é uma técnica apoiada em procedimentos sistemáticos, a qual verifica o conteúdo de textos e indicadores, para inferir, tanto no conhecimento, quanto nas condições de produção e recepção das mensagens.^(^^14^^)^

## RESULTADOS

Utilizaram-se, como base documental, os relatórios divulgados pelas instituições de saúde no ano de 2014. A tabela 1 apresenta a aderência das instituições de saúde aos indicadores da GRI, nos aspectos econômico, social e ambiental.

**Tabela 1:** Aderência das instituições de saúde aos indicadores do *Global Reporting Initiative*

Empresas	Indicadores								

	Econômico			Social			Ambiental		
		
	Adere (%)	Não adere (%)	Não apresentou (%)	Adere (%)	Não adere (%)	Não apresentou (%)	Adere (%)	Não adere (%)	Não apresentou (%)
Hospital-AE	73,5	0,0	26,5	55,6	0,0	44,4	62,5	0,0	37,5
Hospital-AC	32,4	17,6	50,0	33,3	33,3	33,3	35,4	16,7	47,9
Hospital-SL	38,2	0,0	61,8	44,4	0,0	55,6	33,3	0,0	66,7
Média	48,0	5,9	46,1	44,4	11,1	44,4	43,8	5,6	50,7

O percentual expressa o índice de aderência do relatório da instituição financeira em relação ao proposto pela GRI.

A dimensão econômica foi a melhor abarcada pelos indicadores da GRI, com média de aderência de 48,0%. Na sequência, apareceram a dimensão social, com 44,4%, e a ambiental, com 43,8%. O Hospital-AC foi o único a apresentar indicadores não aderentes à GRI nas três dimensões da sustentabilidade. O Hospital-AE apresentou maior aderência em cada uma das três categorias.

A tabela 2 apresenta a classificação dos indicadores sociais das instituições de saúde.

**Tabela 2:** Classificação dos indicadores sociais das instituições de saúde

Indicadores sociais	Hospital-AE	Hospital-AC	Hospital-SL
Avaliação de fornecedores em Direitos Humanos	2	2	-
Avaliação de fornecedores em impactos na sociedade	-	1	-
Avaliação de fornecedores em práticas trabalhistas	-	2	-
Combate à corrupção	3	1	-
Comunidades locais	2	-	2
Diversidade e igualdade de oportunidades	1	1	-
Emprego	3	1	3
Igualdade de remuneração entre mulheres e homens	1	-	-
Investimentos	-	-	1
Mecanismos de queixas e reclamações relacionadas a impactos na sociedade	1	-	-
Mecanismos de queixas e reclamações relacionadas a práticas trabalhistas	1	-	-
Não descriminação	-	-	1
Privacidade do cliente	1	-	-
Relações trabalhistas	1	-	-
Rotulagem de produtos e serviços	3	1	1
Saúde e segurança do cliente	2	1	2
Saúde e segurança no trabalho	4	4	3
Trabalho forçado ou análogo ao escravo	1	-	-
Trabalho infantil	1	-	-
Treinamento e educação	3	3	3

O volume e a diversidade de aspectos foram maiores nas dimensões econômica e ambiental. O Hospital-AE possuía 30 indicadores, enquanto o Hospital-AC tinha 17 e o Hospital-SL, 16. O Hospital-AE foi a instituição que mais aderiu e divulgou seus indicadores sociais, com quase o dobro de indicadores divulgados em relação a seus concorrentes. A classificação dos indicadores ambientais das instituições de saúde está na tabela 3.

**Tabela 3:** Classificação dos indicadores ambientais das instituições de saúde

Indicadores ambientais	Hospital-AE	Hospital-AC	Hospital-SL
Água	3	3	3
Avaliação e ambiente de fornecedores	2	1	-
Efluentes e resíduos	5	3	3
Emissões	7	-	3
Energia	5	3	4
Materiais	2	-	-
Mecanismos de queixas e reclamações relativas a impactos ambientais	1	-	-
Produtos e serviços	-	1	-

A atenção com a dimensão ambiental foi observada em todas as instituições de saúde. O Hospital-AE apresentou a maior quantidade de indicadores (25), destacando-se em relação aos outros dois. O Hospital-SL apareceu em segundo lugar, com 13 indicadores, e o Hospital-AC teve 11. A tabela 4 apresenta a classificação dos indicadores econômicos das instituições de saúde.

**Tabela 4:** Classificação dos indicadores econômicos das instituições de saúde

Indicadores econômicos	Hospital-AE	Hospital-AC	Hospital-SL
Desempenho econômico	4	1	2
Impactos econômicos indiretos	0	2	2
Presença no mercado	1	0	0

O Hospital-AC e o Hospital-SL não apresentaram a presença de mercado, enquanto o Hospital-AE não apresentou os impactos econômicos indiretos. O total de indicadores por instituição de saúde foi: Hospital-AE com 117, Hospital-AC com 85 e Hospital-SL com 69. Considerando-se apenas os indicadores pertencentes à GRI o perfil foi o seguinte: Hospital-AE com 60, Hospital-AC com 33 e Hospital-SL com 31.

O total de indicadores de sustentabilidade em suas três dimensões foi superior no Hospital-AE, enquanto as outras instituições de saúde apareceram próximas em relação ao número de indicadores divulgados. Este perfil mostra maior interesse do Hospital-AE em apresentar seu desempenho na forma de indicadores, de acordo com a recomendação da GRI.

## DISCUSSÃO

O comparativo entre os relatórios de sustentabilidade das três empresas possibilitou a análise combinada dos dados e evidenciou o interesse das instituições de saúde em publicar seu desempenho na forma de indicadores aderentes à GRI. A divulgação dos relatórios corrobora a abordagem do interesse das instituições em divulgar seus dados,^(^^12^^)^ possibilitando aos *stakeholders* entendimento, mesmo que parcial, da sustentabilidade das empresas.^(^^12^^)^

O maior volume de informações divulgadas pelo Hospital-AE pode resultar em benefícios corporativos, principalmente aqueles relacionados à reputação corporativa.^(^^6^^)^ O Hospital-AE iniciou a divulgação de seu relatório em 2006, enquanto os demais hospitais analisados iniciaram este processo 5 anos depois. Este cenário pode ser explicado pela abordagem na qual os concorrentes se sentem pressionados a divulgar seus dados na medida em que um dos *players* destaca-se, por divulgar relatórios que apontam um bom desempenho dos indicadores.^(^^9^^)^ Estar há mais tempo publicando seus indicadores e o maior volume de informações disponíveis do Hospital-AE o posicionam com distinção^(^^4^^)^ e influem em uma melhor reputação da instituição.^(^^5^^)^ Os dados obtidos não possibilitam identificar se o menor volume de informações disponibilizados pelas outras duas instituições está relacionado a razões limitantes.^(^^7^^)^

A curta longevidade de relatórios por parte dos hospitais corrobora o entendimento que aponta a divulgação de desempenho, por parte das empresas, incluindo indicadores sustentáveis como uma ação recente.^(^^8^^)^

As três instituições apresentaram perfis idênticos de proporcionalidade de indicadores. Os indicadores sociais foram os que apareceram em maior volume, seguidos dos ambientais e dos econômicos, condição semelhante à identificada em outras pesquisas.^(^^11^^)^

Observamos conjunto de 20 temas que abarcavam 63 indicadores sociais utilizados pelas empresas. As categorias que se destacaram foram a de saúde e segurança no trabalho, com 11 indicadores, e treinamento e educação, com 9, considerando as três instituições de saúde. Estas categorias tendem a possuir íntima relação entre elas, pois as atividades profissionais desempenhadas demandam a necessidade de atenção à saúde e segurança no trabalho.

Os indicadores ambientais estavam distribuídos em oito categorias, mas concentradas em quatro delas, a saber: energia com 12 indicadores, efluentes e resíduos com 11 indicadores, emissões com 10 indicadores e água com 9 indicadores, considerando as três instituições de saúde. Estes indicadores apontam para dois grupos de atenção: o primeiro voltado à otimização dos recursos (energia e água) e o segundo, ao monitoramento e à mitigação do impacto de suas atividades (efluentes e resíduos e emissões). Isto aponta atenção das instituições de saúde para temas ambientais específicos.

A aderência do Hospital-AE à divulgação de seus indicadores de sustentabilidade se mostrou superior à das demais instituições. O Hospital-AE divulga 117 indicadores, enquanto o Hospital-AC possui 85 e o Hospital-SL com 69. Particularmente sobre os indicadores com aderência ao modelo proposto pela GRI observa-se um predomínio do Hospital-AE, com 63 indicadores aderentes, volume este superior ao somatório dos outros dois hospitais analisados.

## CONCLUSÃO

A comparação das instituições de saúde atuantes no mesmo segmento mostra que houve analogia entre os indicadores adotados pelas empresas. Identificou-se a analogia dos indicadores utilizados pelas três instituições de saúde, tanto para a sustentabilidade ambiental, quanto para a social e a econômica. Adicionalmente, identificou-se a similaridade entre os indicadores utilizados pelas instituições de saúde da dimensão perfil e governança.

Os relatórios de sustentabilidade dos hospitais analisados possuíam indicadores em todas as dimensões da sustentabilidade, no entanto com diferentes intensidades de aderência. Os indicadores da dimensão social apareceram em maior volume e, na sequência, os indicadores ambientais e os econômicos, em menor quantidade.
